# Structure and Properties of Gas-Nitrided, Precipitation-Hardened Martensitic Stainless Steel

**DOI:** 10.3390/ma15030907

**Published:** 2022-01-25

**Authors:** Paweł Kochmański, Marcin Długozima, Jolanta Baranowska

**Affiliations:** 1 Faculty of Mechanical Engineering and Mechatronics, West Pomeranian University of Technology Szczecin, Al. Piastów 19, 70-310 Szczecin, Poland; marcin.dlugozima@secowarwick.com (M.D.); Jolanta.Baranowska@zut.edu.pl (J.B.); 2 Seco/Warwick S.A, Jana III Sobieskiego Str. 8, 66-200 Świebodzin, Poland

**Keywords:** low-temperature nitriding, gas nitriding, precipitation-hardened stainless steel, Nanoflex steel, S phase, expanded austenite, expanded martensite

## Abstract

Nanoflex stainless steel is a promising material for medical applications. However, improvement of its mechanical properties without compromising its corrosion resistance is still a challenge. In order to investigate the effect of the nitriding process on the corrosion and wear resistance of Sandvik Nanoflex^TM^ steel, a number of processes were carried out in a gas atmosphere with differing ammonia contents in the temperature range of 425–475 °C for 4 h. The mechanical properties and wear resistance of the layers were tested using the nanoindentation and pin-on-disc methods, respectively. In order to assess corrosion resistance, potentiodynamic tests were carried out in Ringer’s artificial body fluid and in a 3% aqueous solution of sodium chloride. The results are discussed herein with respect to the microstructural characteristics of the layers studied using light and scanning electron microscopy, X-ray diffraction phase analysis and wavelength dispersive X-ray microanalysis. The structure of nitrided layers included three zones: the subsurface zone composed of nitrides and the zones composed of metastable phases, i.e., the S phase (γN) and expanded martensite (αN) with possible precipitates of nitrides. The third zone adjacent to the steel core was enriched with carbon. The nitrided samples showed significant improvement in the wear rate while maintaining good corrosion resistance in comparison to the non-treated steel. We concluded that nitriding should be carried out at a temperature below 450 °C and in an atmosphere containing no more than approximately 50% ammonia in order to avoid nitrides precipitation.

## 1. Introduction

In this article, we present results of research on nitrided layers of precipitation-hardening stainless steel Sandvik Nanoflex^TM^. This is an ultra-high-strength steel with very good corrosion resistance. Extremely high tensile strength levels (up to 3 GPa for a wire) and high hardness (up to approx. 600 HV) can be achieved by a combination of a cold working, simple heat treatment, i.e., solutioning at approx. 1100 °C, and ageing at 475 °C for 4 h. Achieving such good mechanical properties is possible thanks to very fine nanometric precipitates of intermetallic phases, such as η-Ni_3_(Ti, Al), Fe_2_Mo, ε-Cu and R [1,2,3,4,5,6], precipitating in martensitic matrix. Due to its special properties, this steel is suitable for production of durable mechanical parts, such as turbine blades and bearings. Medicine is also a significant field of application for Nanoflex steel, and it is used for manufacturing of various skin-piercing applications (surgical staples, retractable syringes and needles for vaccination and acupuncture) and surgical or microsurgical instruments (including blades, saws, bone drills, biopsy punches and screwdrivers).

Intensive precipitation of chromium nitrides and iron nitrides in the diffusion zone in austenitic stainless steels and martensitic stainless steel makes gas nitriding at standard temperatures (>500 °C) unfavourable. This is because despite the increase in hardness and abrasion resistance, the corrosion resistance of this type of steel is significantly reduced due to the appearance of precipitates of chromium nitrides and simultaneous depletion of Cr in the matrix [7]. When the process is carried out at a temperature below 450 °C, a metastable S phase called expanded austenite can be observed in austenitic stainless steel. This phase is actually a supersaturated solution of nitrogen in austenite and has very good corrosion resistance and high hardness (around 1500 HV) [7,8,9,10,11,12]. In martensitic stainless steels, the phase that forms in low-temperature nitriding is called expanded martensite [13] (also referred to as expanded ferrite), which is, in fact, a supersaturated interstitial solid solution of nitrogen in the martensitic phase, providing high hardness, high sliding wear resistance and increased fatigue and corrosion resistance, which makes low-temperature treatment advantageous [14,15].

A well-known technological challenge is that additional surface activation is required to perform the gas-nitriding process on all stainless steels. A tight layer of chromium oxides forms on the stainless steel surface, which hinders the diffusion of nitrogen atoms into the steel, especially at lower temperatures [16]. There are many methods available and in use for surface activation of stainless steels. Often used are so-called chemical activation methods, among which the following can be distinguished: liquid and solid chemicals containing chlorine compounds, such as CCl_4_, NH_4_Cl, polyvinyl chloride; fluorine compounds, such as NF_3_, hydrocarbons or other gases containing carbon and nitrogen; and phosphating or pre-oxidation [9,17,18,19]. The other activation methods are based on mechanical activation, such as grinding, as well as physical activation by cathode sputtering and bombarding the surface with low-energy ions. The latter usually forms part of ion nitriding but can also be used as an activating pre-treatment prior to gas nitriding [9].

Limited research has been conducted to examine the gas- and plasma-nitrided layers on other precipitation-hardening stainless steel, i.e., 17-4 PH [20,21,22,23,24,25] or Corrax [26], and few studies have been conducted on the structure of nitrided layers on Nanoflex steel [26,27,28,29]. The structure of low-temperature nitrided layers described in previous works [26,29] was composed mainly of expanded martensite and the S phase. The presence of the S phase was observed only in layers produced on Nanoflex steel containing significant amounts of austenite, contrary to layers on cold worked steel, where the layers were composed exclusively of expanded martensite. Nitrogen is a strong stabilizer of the austenitic structure in stainless steels, and in [28], the presence of significant amounts of S phase was observed in layers nitrided at a high nitriding potential on Nanoflex steel containing a negligible fraction of residual austenite.

There are literature studies on the corrosion and wear properties of nitrided layers on martensitic stainless steels hardened by carbon [7,14,15,30,31,32,33,34,35,36,37,38] and 17-4 PH [24,39] steel, but the data on the performance of nitrided Nanoflex steel are practically unavailable.

The objectives of this work were: production nitrided layers on commercially available Sandvik Nanoflex^TM^ steel in the condition of maximum hardness, determination of an influence of the nitriding parameters on the layer structure, as well as corrosion and wear properties. As Nanoflex steel is used in the production of medical instruments, it was important to determine the corrosion resistance in the environment of body fluids. Therefore, corrosion tests were performed in Ringer’s type solution.

## 2. Materials and Methods

### 2.1. Protocol for Production of the Nitrided Diffusion Layers

The substrate used in the experiment was Nanoflex steel (Sandvik AB, Sandviken, Sweden) of chemical composition given in Table 1. The steel was nitrided under three technological conditions denoted as: A, B, C. Technological operations performed under these conditions and the corresponding hardness values are listed in Table 2. The nitriding kinetics and the structure of the layers produced under conditions A and B were described in previous works [27,28,40].

Before nitriding, the samples of dimensions 20 mm × 10 mm × 1.5 mm were mechanically ground and polished to a roughness of Ra = 0.05 µm. Electropolishing was used as the final surface preparation, and it was conducted in a mixture of diethyl ether: 175 mL, ethanol: 75 mL and perchloric acid: 100 mL, with the following parameters: temperature: −18 °C; time: 60 s; current density: c.a. 0.2 A/cm^2^; and voltage: 9 V. The samples were cleaned ultrasonically in isopropyl alcohol prior to nitriding.

The surface treatment was conducted using non-commercial equipment constructed at the West Pomeranian University of Technology (Szczecin, Poland) [28]. The surface treatment consisted of two steps: ex situ physical activation (removal of chromium oxides to enable nitrogen diffusion) and gas nitriding. Hydrogen plasma sputtering was applied as the activation method. Sputtering was conducted in a separate chamber connected via a canal lock with a quartz retort with a diameter of 150 mm and a length 1 m. A quartz tube was used to avoid ammonia dissociation onto the walls of the retort. Before the ion sputtering process, the activation chamber was evacuated with a rotary vacuum pump to a pressure level of 0.2 Pa and then purged with hydrogen. The gas pressure during ion sputtering was altered within a the range from 2 to 7 Pa to maintain the required value of current density from 0.5 to 3.5 mA/cm^2^. The duration of the activation process was 15 min. A direct current (DC) power generator operating at a voltage of 1.3 kV was used for this purpose.

After the activation step, the samples were transferred through the canal lock into the retort, where the required gas-treatment parameters of temperature and gas mixture were already established. The temperature of the furnace was controlled with an accuracy of ±2 °C. The degree of ammonia dissociation was measured in the exhaust gas with a dissociation pipette. The working atmosphere consisted either of pure ammonia or a mixture of ammonia and products of its dissociation obtained by external thermal-dissociation equipment. The gas-nitriding processes were conducted with parameters given in Table 3.

### 2.2. Characterization Protocol

After mechanical cutting, the samples were electrochemically nickel-plated to protect the nitrided edges during further metallographic preparation. The samples were mounted in a conductive resin (Polyfast, Struers, Ballerup, Denmark) and mechanically ground and polished using a 9, 3, 1 and 0.25 µm diamond suspension. The final stage of the sample-preparation procedure was low-angle (c.a. 10°) argon ion milling (Flat Milling System IM-3000, Hitachi, Naka, Japan). Microstructures on the cross sections for light-microscopy examinations were chemically revealed using Mi19Fe etchant containing 3 g of ferric chloride, 10 cm^3^ hydrochloric acid and 90 cm^3^ ethanol.

The diffusion layers were examined using an FE-SEM SU-70 (Field Emission Scanning Electron Microscopy) microscope (Hitachi, Naka, Japan) and WDS (wavelength dispersive spectrometry)/EDS (energy dispersive spectrometry) X-ray microanalysis using the NORAN™ System 7 from Thermo Fisher Scientific (Madison, WI USA). The latter consisted of Magnaray (WDS), UltraDry X-ray detector (EDS) integrated components. The WDS analysis was performed at an accelerating voltage of 10 kV and an electron-beam current of approximately 20 nA using CrSc80 diffracting crystal for nitrogen. The WDS quantitative analytical procedure was based on Cr_2_N standard. Net-count elemental mappings were acquired with a resolution of 1024 by 768 and a pixel size of 0.04 µm. Quantitative profiles of nitrogen and carbon concentrations in the layers were derived from the WDS point analysis. For each position of the distance from the surface, three analyses were performed, and the average values are presented in the graphs. The “PROZA” [41] correction method was applied for WDS quantitative analysis, and the estimated standard uncertainty for the WDS measurements was 0.05 wt.%. All X-ray microanalysis examinations were performed on flat (not etched) sample surfaces.

Light microscopy (LM) examinations were performed using a Nikon Ephiphot 200 (Nikon Corporation., Tokyo, Japan).

X-ray diffraction (XRD) phase analysis was performed using X-ray tube CoKα, operating at parameters of 35 kV, 45 mA, as well as a Bragg–Brentano geometry (X’Pert–PRO, Panalytical, Almelo, The Netherlands). The applied step of the goniometer was 0.05°, and the acquisition time was 200 s. The acquired data were processed using X’Pert HighScore (v. 2.2.1) software provided by Panalytical (The Netherlands). 

Hardness measurements were performed with Vickers indenter (LV700AT, Leco, St. Joseph, MI, USA).

The distribution profiles of selected chemical elements were acquired using glow-discharge optical emission spectrometry (GDOES) (Horiba Jobin Yvon, Montpellier, France).

Corrosion tests were performed with a potentiometric method (Atlas 9833, Atlas-Sollich, Gdańsk, Poland) at room temperature in a flat cell, which was a three-electrode setup consisting of a saturated calomel reference electrode, a platinum counter electrode and a working electrode (sample). The sample to be tested was placed against a Teflon^®^ ring at one end of the flat cell, leaving an area of 0.38 cm^2^ on the sample surface in contact with the testing solution through a round hole in the Teflon^®^ ring sealing. Two types of solution were used for the tests, i.e., Ringer’s solution and 3% NaCl in distilled water. During the corrosion test, the solution had not been degassed. The potential range of –1500 to +2000 mV was scanned at a rate of 5 mV/s.

Nanoindentation measurements were performed using Nano Indenter XP (MTS, Oak Ridge, TN, USA) at an increasing load from 0 to 0.7 N with a Berkovich tip.

Tribological tests were performed on a TRN S/N 18−324 Pin-on-Disk Tribometer (CSM Instruments, Peseux, Switzerland) in an accordance with standard ASTM G 99. The tests were conducted at an air temperature of 22–24 °C and a relative humidity of 48–50%. An alumina ball with a diameter of 6 mm was used as a counterprobe loaded with 3 N. The value of the load was calculated in such a way that the maximum stress would not exceed the yield point of the hardened steel. The reciprocating movement with a stoke of 8 mm was applied. The distance and maximum linear speed of the tests were 152 m and 30 mm/s, respectively. The wear rate was calculated using Formula (1).

(1) $$K=\frac{V}{F\cdot S}$$

where:

*K*—wear rate $\left[\frac{\mu m^{3}}{N\cdot m}\right]$,
*V*—total volume loss [μm^3^], *V* = *V*_sampe_ + *V*_ball_,
*V*_sample_—loss of sample’s material volume [μm^3^],
*V*_ball_—loss of countersample’s material (ball) volume [μm^3^],
*F*—load [N],
*S*—distance [m]

The loss of a sample’s material volume was determined on the basis of profilometric (stylus profiler Dektak 6M, Veeco Instruments Inc., Tucson, AZ, USA) and microscopic measurements (measuring microscope, Nikon MM-40, Nikon Corporation., Tokyo, Japan).

## 3. Results and Discussion

### 3.1. Structural Characterization

**Figure 1:**
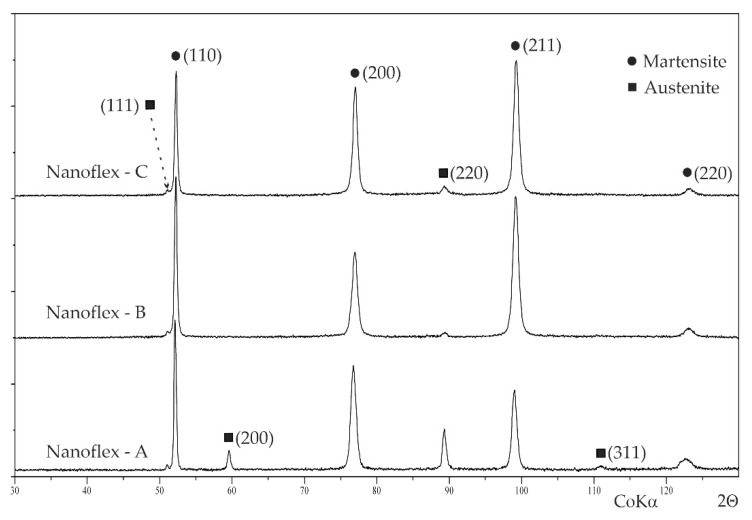
XRD patterns acquired from Nanoflex steel under various technological conditions.

XRD patterns acquired from Nanoflex steel under conditions A, B and C (in an accordance with Table 2) are shown in Figure 1. Generally, all samples, regardless technological conditions, were composed of two major phases, i.e., martensite (b.c.c. structure) and a certain amount of retained austenite (f.c.c. structure). The quantity of retained austenite in the steel under condition A (after solutioning) was significantly greater in comparison with the other conditions due to strain-induced martensitic transformation. For all technological conditions, comparing the intensities of peaks indexed as (110), (200) and (211) for martensite and (111) and (220) for austenite, it can be concluded that the steel had a dominant texture produced by cold working. In the XRD pattern acquired from the steel under condition C (after ageing), no peaks from the strengthening phases (obviously present in the structure [2,3,42]) were visible due to their volume being too low.

**Figure 2:**
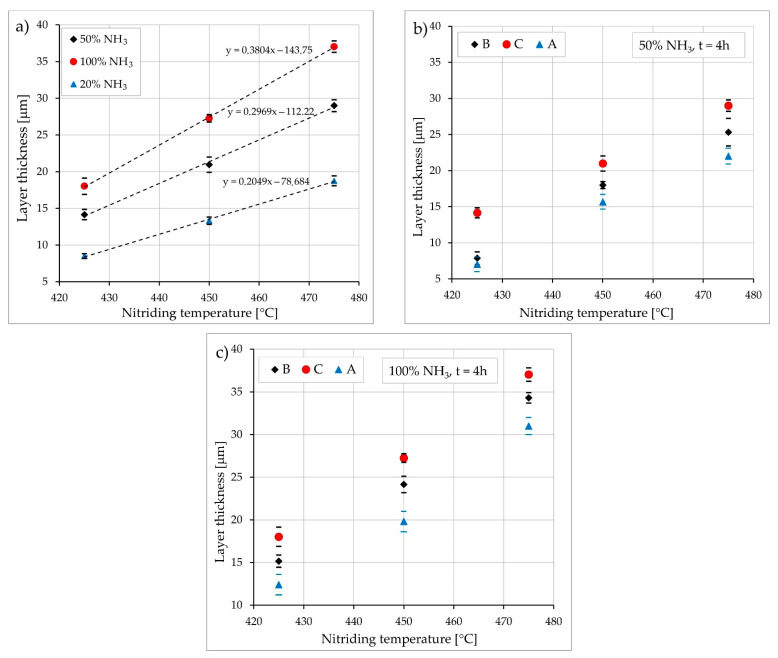
Thickness of nitrided layers in the function of nitriding temperature: (**a**)—Nanoflex steel under condition C; (**b**) and (**c**)—Nanoflex steel under conditions A, B and C.

Results of thickness measurements are presented in Figure 2. It is clearly visible that, as expected, the increase both in the temperature and the amount of ammonia in the working atmosphere had a strong positive effect on the growth kinetics of nitrided layers, which could be due to the influence of the technological condition of the steel before nitriding. The thinnest layers were obtained on the steel under condition A, and the thickest layers were obtained under condition C (Figure 2b,c) for all temperatures and nitriding potentials (amount of ammonia). The slowest growth of the layers was observed on the steel containing a significant amount of austenite (condition A), which is characterized by a lower diffusion coefficient of interstitial elements compared to ferrite and martensite. The thickest nitrided layers were obtained in the case of the Nanoflex steel after ageing, which could be explained by the presence of numerous very fine precipitates of the strengthening phases, which could multiply the quantity of grain boundaries, i.e., paths of easy diffusion.

**Figure 3:**
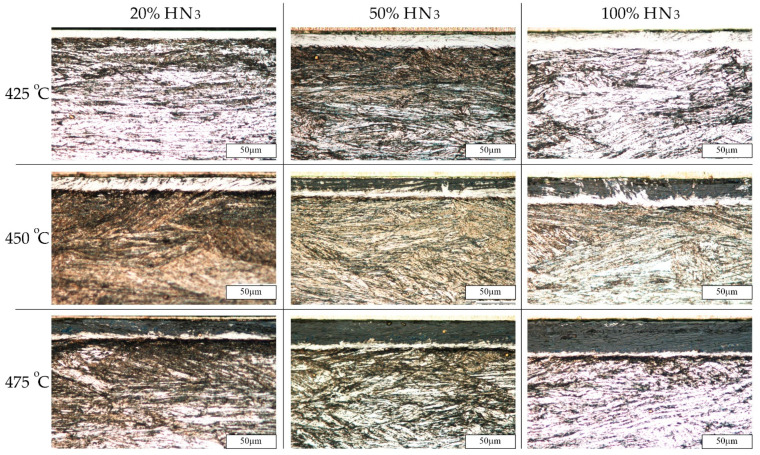
Cross sections of nitrided layers obtained at various temperatures and atmospheres with a constant nitriding time of 4 h. Light microscopy.

**Figure 4:**
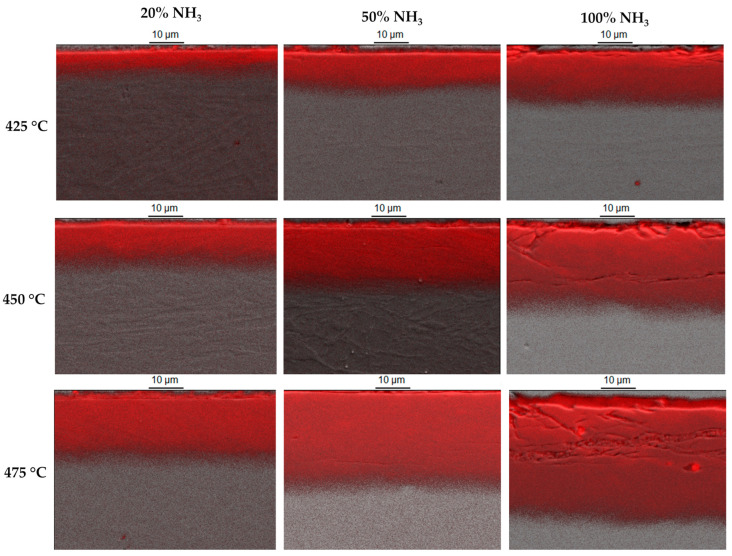
Qualitative distribution of nitrogen (red colour) on cross sections of nitrided layers on Nanoflex steel under condition C with various treatment parameters (treatment time: 4 h). Secondary electron image and WDS X-ray microanalysis.

Light-microscopy photographs of microstructures of the nitrided layers are shown in Figure 3. The thickness of all layers was uniform on the entire surface of the samples, regardless of the nitriding conditions. The layers produced under different technological parameters demonstrated distinct susceptibilities to chemical etching. The layers obtained at the lowest temperature and with the least amount of ammonia in the atmosphere were white and much more resistant to etching with Mi19Fe etchant in comparison to layers nitrided at 450 and 475 °C or compared to layers nitrided in 50% and 100% ammonia atmospheres. As the nitriding temperature increased, the dark area in the layer structure grew. This temperature effect is more pronounced for a higher nitriding potential (atmospheres with ammonia amount of 50 and 100%). The dark areas in the layer microstructure may suggest the presence of nitrides that change (deteriorate) the corrosive properties of the steel [7,36]. All layers contained a nitride sublayer close to the surface. The sublayer was visible in the scanning electron microscope photographs (Figure 4) of the cross-sections of the layers, except for the layer obtained at 425 °C in an atmosphere of 20% ammonia. The described microstructure differs significantly from the needle-like microstructure observed by the authors in the case of nitrided layers on Nanoflex steel under condition A [27,43]. In the case of nitrided layers at 450 and 475 °C in an atmosphere of 100% ammonia, microcracks parallel to the surface were observed (Figure 4), which may indicate high residual stress resulting in embrittlement of the layers. A high residual-stress value generated by nitriding was observed in layers on 17-4 PH precipitation-hardened stainless steel [23].

**Figure 5:**
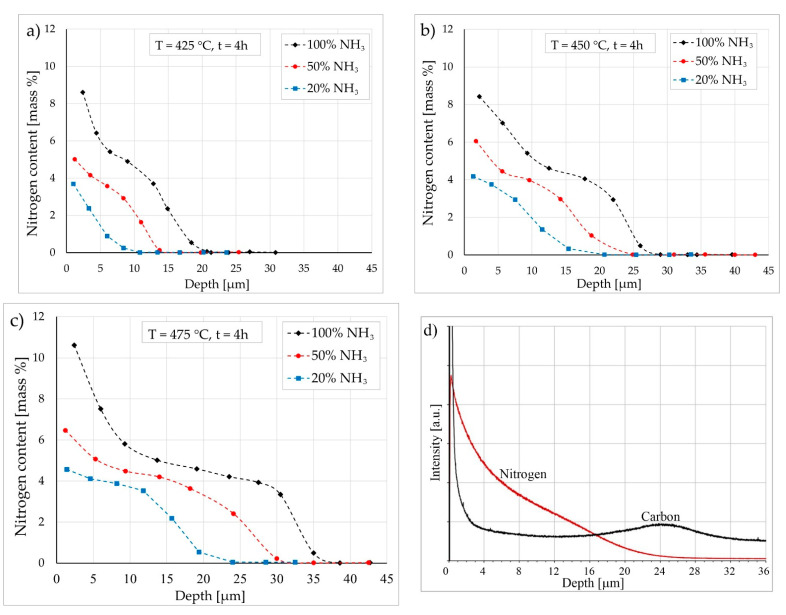
(**a**–**c**) Quantitative distribution of nitrogen on cross sections of nitrided layers on Nanoflex steel under condition C with various treatment parameters (treatment time: 4 h), WDS X-ray microanalysis. (**d**) Qualitative distribution of nitrogen and carbon on cross sections of nitrided layer (450 °C, 50% NH_3_, 4 h) on Nanoflex steel under condition C, GDOES.

The quantitative WDS X-ray microanalysis profiles for nitrogen distribution on the layer cross sections are presented in Figure 5, consistent with the qualitative WDS maps (Figure 4).

The maximum content of nitrogen (approx. 10 wt.%) was detected for layers produced in an atmosphere containing 100% ammonia at a temperature of 475 °C (Figure 5c). The minimum nitrogen content (below 4 wt.%) was detected in the case of an atmosphere of 20% ammonia at 425 °C (Figure 5a). The typical profile of carbon on layer cross sections is shown in Figure 5d. A sublayer of increased carbon content was present below nitrided layers. Such a sublayer was reported in low-temperature nitrided or nitrocarburized layers on austenitic stainless steels; however, the root cause of such a shape of the carbon profile in diffusion layers has not yet been convincingly explained in the literature [44].

**Figure 6:**
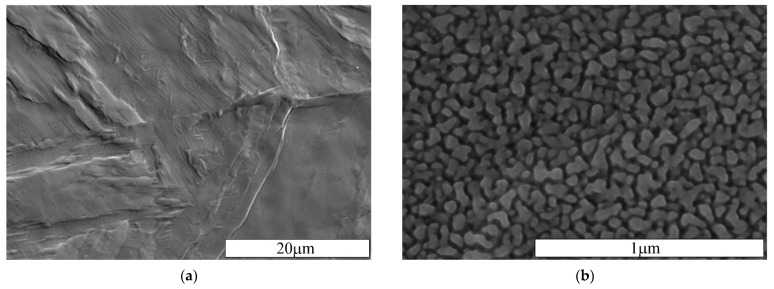
Secondary electron images of the surface of the nitrided layer at a temperature of 425 °C for 4 h in an atmosphere containing 20% NH_3_; (**a**) surface relief, (**b**) surface nitrides.

A characteristic surface relief, as shown in Figure 6a, was caused by nitriding, regardless of technological parameters. This relief reflects the needle-like martensitic nature of the steel structure and shows traces of slip bands—twins demonstrating the significant stresses caused by nitriding. An increase in surface roughness was observed, i.e., arithmetical mean deviation of the profile (Ra), after nitriding rose to c.a. 0.25 ± 0.05 µm. This phenomenon is typical for gas low-temperature thermochemical treatment of stainless steels and was also observed by the authors on nitrocarburized austenitic steel [44]. The presence of a sublayer containing nanometric nitrides was confirmed by high-resolution scanning electron microscopy of the surface (Figure 6b). The sublayer was observed for all the layers, including obtained at 425 °C in an atmosphere containing 20% NH_3_.

**Figure 7:**
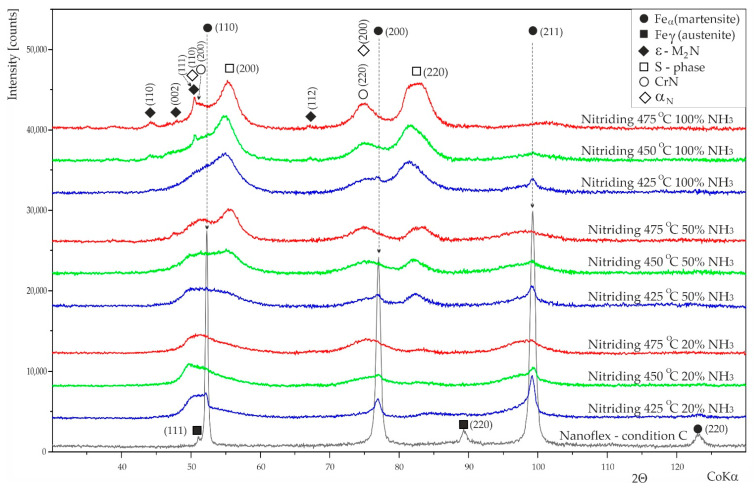
X-ray diffraction patterns of nitrided layers with various parameters and untreated Nanoflex steel in condition C. (Intensity of every next pattern has been shifted up 4000 counts from the previous pattern).

The X-ray diffraction phase analysis results are shown in Figure 7. Interpretation of the diffraction patterns acquired from the nitrided layers might be difficult due to low-intensity, broadening peaks.

For the layers produced in an atmosphere containing 20% ammonia, no sharp peaks were observed in the diffraction patterns, except for those from the substrate. Diffraction peaks from the steel substrate were observed for all samples nitrided at temperature of 425 °C in all types of atmospheres and in layers produced at 450 °C in atmospheres containing 20 and 50% NH_3_ due to their low thickness and the use of deeply penetrating X-ray with CoKα characteristic wavelengths.

The shape of the diffraction patterns indicates that both expanded austenite (also referred to as S phase) and nitrogen-expanded martensite were present in the structure of the layers and constitute the main phase components. Usually, the intensity is expanded; however, martensite peaks are very low and broad. Thus, the shape of diffraction patterns indicates the presence of severe defects in the crystal structure.

Based on XRD, it is difficult to confirm the presence of nitrides in layers produced in atmospheres containing 20 and 50% NH_3_. However, a low-intensity, broad “peak”, which could be attributed to CrN, was observed in the layers treated at temperatures above 450 °C. On the other hand, the occurrence of nitrides was confirmed with microstructural observations on the cross sections after etching (Figure 3). Moreover, SEM examinations of the surface revealed the presence of very fine nitrides (Figure 6).

For layers produced in an atmosphere of 100% ammonia, significantly higher intensities for S-phase reflections and sharper peaks attributed to ε-type nitrides ((Fe, Cr)_2_N) were visible for layers nitrided at temperature above 450 °C. The presence of CrN nitrides is also very probable; however, the diffraction peak from planes (200) of the theoretical intensity of 100% overlaps with other phase components. The presence of CrN in nitrided layers on other grades of martensitic stainless steel was reported in [45]. The intensities of the peaks from ε and CrN phases increase with the treatment temperature. The number of nitrides visible in the diffraction patterns was well correlated with the etching resistance observed in the light-microscopy photographs (Figure 3).

The peaks assigned to the S phase were shifted towards the two lower theta angles, suggesting a larger lattice parameter compared to layers nitrided in atmospheres with lower ammonia content due to the higher nitrogen content in this phase.

The layers nitrided in atmospheres with lower ammonia content, i.e., 20 and 50%, were composed mainly of expanded martensite, while S phase was a major phase component of the layers obtained a 100% ammonia atmosphere. This could be explained by the achievement of the maximum solubility of nitrogen in expanded martensite, and consequently, the driving force for the development of the S phase was generated [26]. The tendency to form an S phase could be enhanced by the presence of larger amounts of austenite in the initial steel structure [27,28].

### 3.2. Properties of the Layers

**Figure 8:**
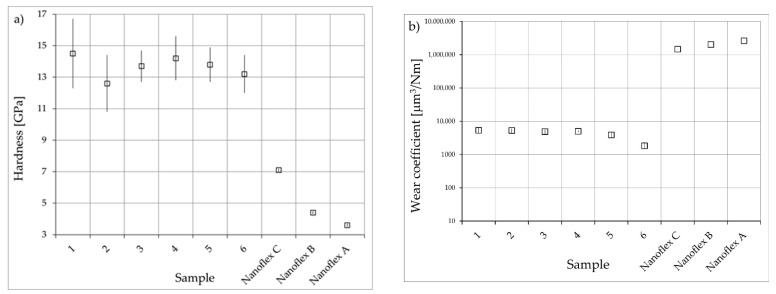
(**a**) Hardness, (**b**) wear coefficient of nitrided layers and Nanoflex steel under conditions A, B and C.

The results of hardness measurements derived from nanoindentation tests are presented in Figure 8a. The hardness of all tested nitrided layers was similar, i.e., in the range of approx. 13 to 15 GPa, which corresponds to approx. 1300 to 1500 HV, while the hardness of the steel substrate was 3.6, 4.4 and 7.1 GPa under technological conditions A, B and C, respectively.

The results of the tests of resistance to tribological wear showed a significant increase (approx. 275 times) caused by nitriding under dry-friction conditions (Figure 8b) in comparison with the hardest condition (C) of the Nanoflex steel. There were no significant differences in the values of the K wear coefficient between different types of nitrided layers, except for the layer produced at a temperature of 475 °C in an atmosphere containing 50% ammonia (sample No. 6), for which the wear rate was evidently lower, possibly due to relatively greater thickness and absence of cracks in the microstructure (Figure 4).

**Figure 9:**
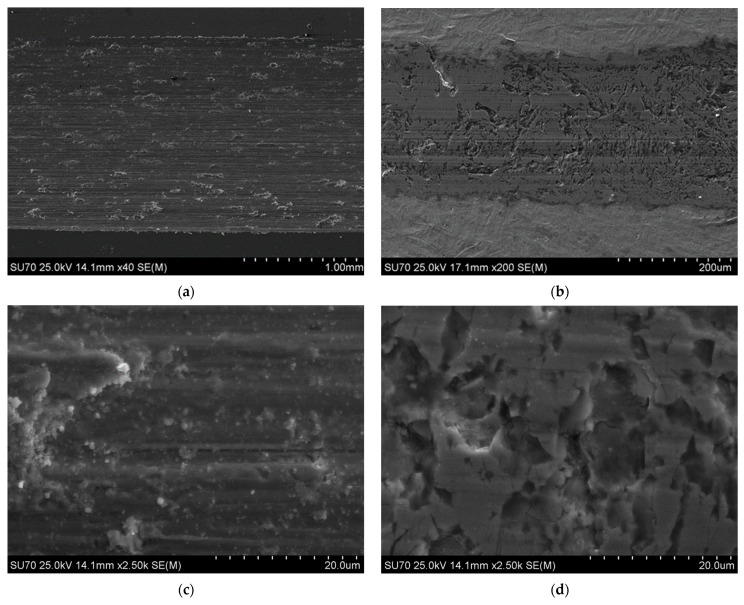
Secondary electron images of the surface of wear tracks after tribological tests of: (**a**,**c**) Nanoflex steel in condition C, (**b**,**d**) layers nitrided at a temperature 450 °C for 4 h in an atmosphere containing 100% NH_3_.

SEM microscopic observations of the surface allowed for the evaluation of the tribological wear mechanism. In the case of non-nitrided Nanoflex steel under all technological conditions and dry friction, the tearing of material particles typical of the adhesive wear mechanism was observed. The surface of the wear track of non-nitrided Nanoflex steel under condition C is shown in Figure 9a,c.

The surface of the wear tracks observed on the nitrided layers after dry tests (Figure 9b,d) proves that the dominant tribological wear mechanism was abrasive wear and scuffing. However, microcracks were also observed, which may indicate increased brittleness of the layers, in particular those produced in an atmosphere of 100% ammonia, which is consistent with the results of microstructural examinations of cross sections. Increased embrittlement of the layers produced in an ammonia-rich atmosphere could be caused by the high residual stress and the presence of nitride precipitates.

**Figure 10:**
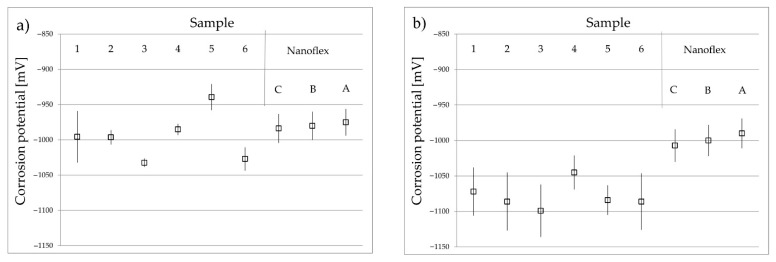
Values of corrosion potentials for nitrided layers and Nanoflex steel under conditions A, B and C. (**a**) Test in Ringer’s solution, (**b**) test in 3% NaCl in water.

The analysis of the results of the potentiodynamic corrosion-resistance tests a Ringer’s fluid environment and, more specifically, the values of the corrosion potential (Figure 10) showed that nitriding at temperatures of 425 and 450 °C did not significantly deteriorate this resistance in relation to the Nanoflex steel without a layer under all conditions (A, B and C); all obtained values were comparable with one another. In the case of tests in an environment of 3% aqueous sodium chloride solution, the values of the corrosion potential in the case of nitrided layers were several tens mV lower than the value measured for the untreated Nanoflex steel, with the corrosion current density (not presented in this work) at the same level. For the layers nitrided at a temperature of 475 °C (samples no. 3 and 6), the values of the corrosion potential were lower compared to the lower treatment temperatures for both test fluids. This indicates a certain deterioration in corrosion resistance caused by the presence of nitrides in the structure of the layers, which was confirmed by metallographic and XRD examinations.

**Figure 11:**
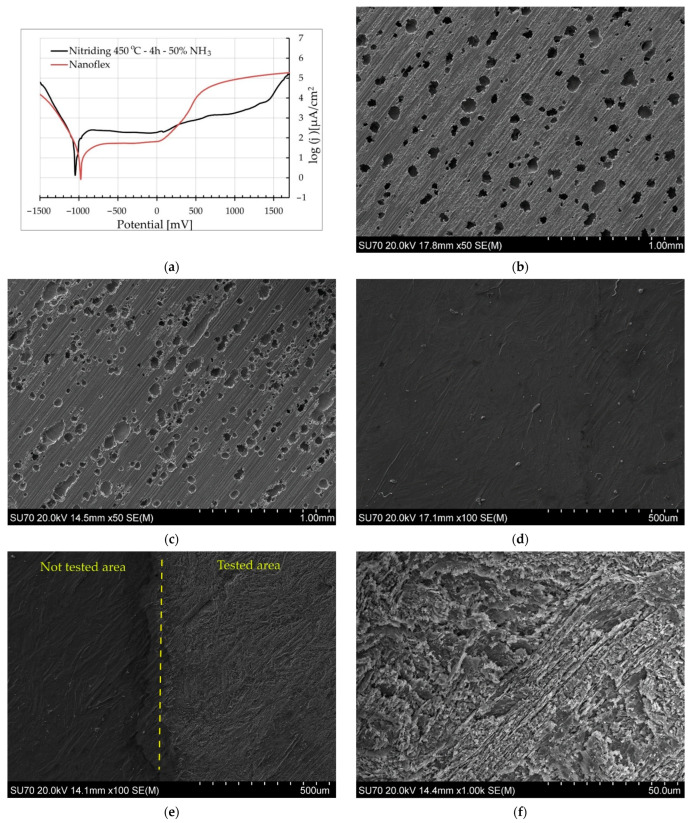
(**a**) Potentiodynamic polarization graphs (in Ringer’s solution) for Nanoflex steel under condition C and nitrided layer (450 °C, 4 h, 50% NH_3_); (**b**–**f**) secondary electron images of surfaces after potentiodynamic corrosion tests of: (**b**) Nanoflex steel tested in 3% NaCl in water, (**c**) Nanoflex steel tested in Ringer’s solution, (**d**) nitrided layer (450 °C, 4 h, 50% NH_3_) tested in Ringer’s solution, (**e**,**f**) nitrided layer (475 °C, 4 h, 100% NH_3_) tested in Ringer’s solution.

The shape of the polarization curves collected during measurements in Ringer’s fluid (Figure 11a) for steel without a layer differed from that obtained for nitrided layers. For the latter, a much larger passive range was observed, without an increase in the current density, compared to the curve acquired from untreated Nanoflex steel, for which a strong increase in the current density was visible after the applied potential exceeded the zero value. The shape of polarization curves observed in the case of nitrided layers indicates increased resistance to pitting corrosion. A strong increase in the current density was recorded at the pitting corrosion potential, usually above 1 V. There were no significant differences in the shape of polarization curves obtained for the nitrided layers produced with the different technological parameters of nitriding. Similar relationships were observed in the case of tests in an aqueous solution of sodium chloride, for which the values of the pitting potential were also above 1 V for the nitrided materials, while for Nanoflex steel without a layer, the pitting-potential value was approximately 100 mV.

For a more complete analysis of the corrosion resistance of the nitrided layers, SEM observations of the surfaces were performed after polarization tests. In the case of Nanoflex steel, the surface after polarization in the environment of Ringer’s fluid (Figure 11b,c) was strongly corroded with numerous pits. The Nanoflex steel samples without the diffusion layer, after polarization tests in sodium chloride solution, had a less changed surface but with equally numerous corrosion pits.

The samples with nitrided layers in an atmosphere containing 50% NH_3_, regardless of the process temperature in the range of up to 475 °C, showed slightly changed surface morphology after polarization (Figure 11d). The polarization did not cause pitting corrosion on the surface, which confirms the greater tendency to passivation of nitrided layers compared to untreated steel.

In the case of nitrided layers in an atmosphere of 100% ammonia, significant corrosion changes in the surface morphology were observed after polarization tests (Figure 11e,f). The surface exposed to the test (Figure 11f) had a needle-like character but without corrosion pits, even for layers nitrided at a temperature of 475 °C. Corrosion changes had a uniform character, in contrast to pitting. Nevertheless, it should be stated that the corrosion resistance of layers nitrided in an atmosphere with high nitriding potential was inferior to that of the layers produced in an atmosphere with lower nitriding potential.

## 4. Summary

**Figure 12:**
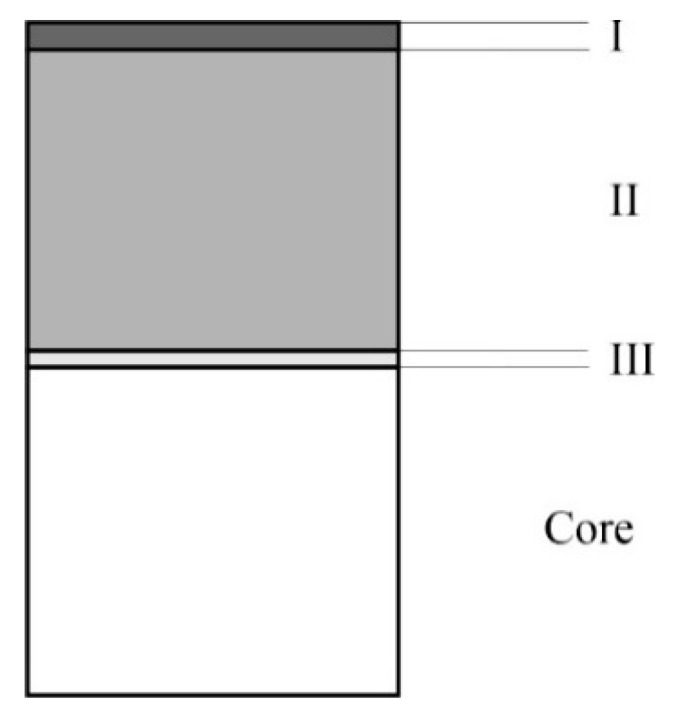
Scheme of microstructure of gas-nitrided layers on Nanoflex precipitation-hardening stainless steel.

The structure of all layers produced in this experiment can be schematically presented in accordance with Figure 12; three zones could be distinguished. The subsurface, zone I, is the zone of nitrides, usually of the ε type. The second thickest, zone II, is the zone composed of the metastable phases: S phase (γN) and expanded martensite (αN) with precipitates of MN-type nitrides (most often CrN). Zone III, adjacent to the steel core, was enriched with carbon. The proportions of the γN and αN phases in zone II depend on the parameters of nitriding—in particular, on the nitrogen potential and the content of residual austenite in the initial structure of the Nanoflex steel. An increase in the nitriding potential and the residual austenite content facilitates the formation of the γN phase. The amount of nitride precipitates in the matrix of the γN and αN phases depends mainly on the nitriding temperature (increasing the nitriding temperature promotes the precipitation of nitrides) but also on the nitriding potential of the working atmosphere, the effect of which is similar to the increasing temperature but not as strong.

All the produced layers, regardless of the technological parameters of nitriding, were characterized by high hardness, at least twice as high as the hardness of Nanoflex steel under the condition of the highest hardening (condition C). No significant influence of the technological parameters of the layer production on the mechanical properties was observed.

Nitriding significantly increased resistance to tribological wear under dry-friction conditions; the wear coefficient decreased by more than 200 times compared to the steel without a layer, which was caused by an increase in hardness and the reduction in adhesive wear.

The corrosion resistance of the nitrided layers under the conditions of Ringer’s fluid and aqueous sodium chloride solution was not deteriorated. Furthermore, the tendency of the steel to passivation increased. Increasing the nitriding temperature to above 450 °C caused a loss of corrosion resistance due to intensively precipitating nitrides and decomposition of the metastable S phase, as well as expanded martensite.

Nitriding of Nanoflex steel is a promising route to significantly improve its mechanical properties (hardness and wear resistance). However, considering the obtained test results, it has to be stated that nitriding should be carried out at a temperature below 450 °C and in an atmosphere containing no more than approximately 50% ammonia in order to avoid precipitation of nitrides.

## Figures and Tables

**Table 1:** Chemical composition of Nanoflex steel [wt.%].

C	Si	Mn	Cr	Ni	Mo	Cu	Ti	Al	Fe
0.02	0.25	0.16	12.68	9.25	3.51	1.97	0.87	0.22	Bal.

**Table 2:** Nanoflex steel conditions and corresponding Vickers hardness.

Condition	Technological Operations	Vickers Hardness HV_30_
A	Solutioning: temperature: 1120 °C/time: 1 h/vacuum atmosphere/nitrogen quenching	271 ± 7
B	Solutioning/cold working	375 ± 7
C	Solutioning/cold working/ageing: 475 °C/time: 4 h/air atmosphere	602 ± 11

**Table 3:** Nitriding parameters used in the experiment and sample denotation.

Sample	Temperature (°C)	Atmosphere Composition (vol.%)		Duration (h)
		NH_3_	Dissociated NH_3_	
1	425	100	0	4
2	450			
3	475			
4	425	50	50	
5	450			
6	475			
7	425	20	80	
8	450			
9	475			

## Data Availability

Data are contained within the article.
